# Supplementary material for: Hemangiosarcoma cells induce M2 polarization and PD-L1 expression in macrophages
Source: Sci Rep. 2022 Feb 8;12:2124. doi: 10.1038/s41598-022-06203-w (PMC8826392; doi:10.1038/s41598-022-06203-w)
Supplement: Supplementary file 1 — Supplementary Information. [file 41598_2022_6203_MOESM1_ESM.pdf]

Supplementary files for

Hemangiosarcoma cells induce M2 polarization and PD-L1 expression in macrophages

Kevin Christian M. Gulay[1], Keisuke Aoshima[1*], Naoya Maekawa[2], Tamami Suzuki[1], Satoru Konnai[2,3], Atsushi Kobayashi[1] & Takashi Kimura[1]

[1] Laboratory of Comparative Pathology, Department of Clinical Sciences, Faculty of Veterinary Medicine, Hokkaido University, Sapporo, Hokkaido, 060-0818, Japan.
[2] Department of Advanced Pharmaceutics, Faculty of Veterinary Medicine, Hokkaido University, Sapporo, Hokkaido, 060-0818, Japan.
[3]Laboratory of Infectious Diseases, Department of Disease Control, Faculty of Veterinary Medicine, Hokkaido University, Sapporo, Hokkaido, 060-0818, Japan.

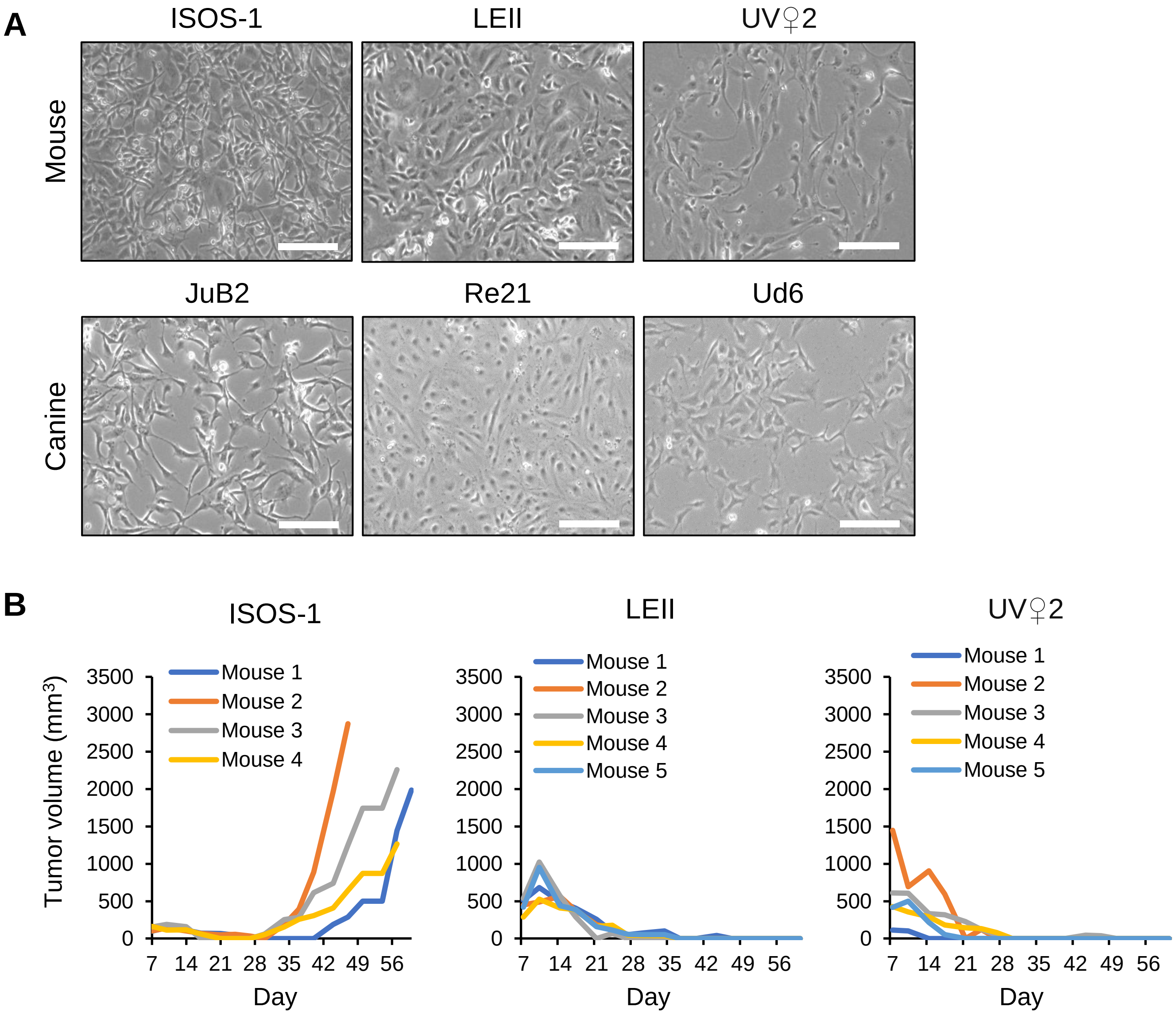

Supplementary figure 1. Morphologies of mouse and canine HSA cell lines and tumor growth curves of mouse HSA cell lines.

A, Phase-contrast images of mouse HSA cells (ISOS-1, UV♀2), mouse immortalized endothelial cells (LEII), and canine HSA cell lines (JuB2, Re12, Ud6). **B,** Tumor growth curves of ISOS-1, LEII and UV♀2 in Balb/c mice. Bars = 125μm.

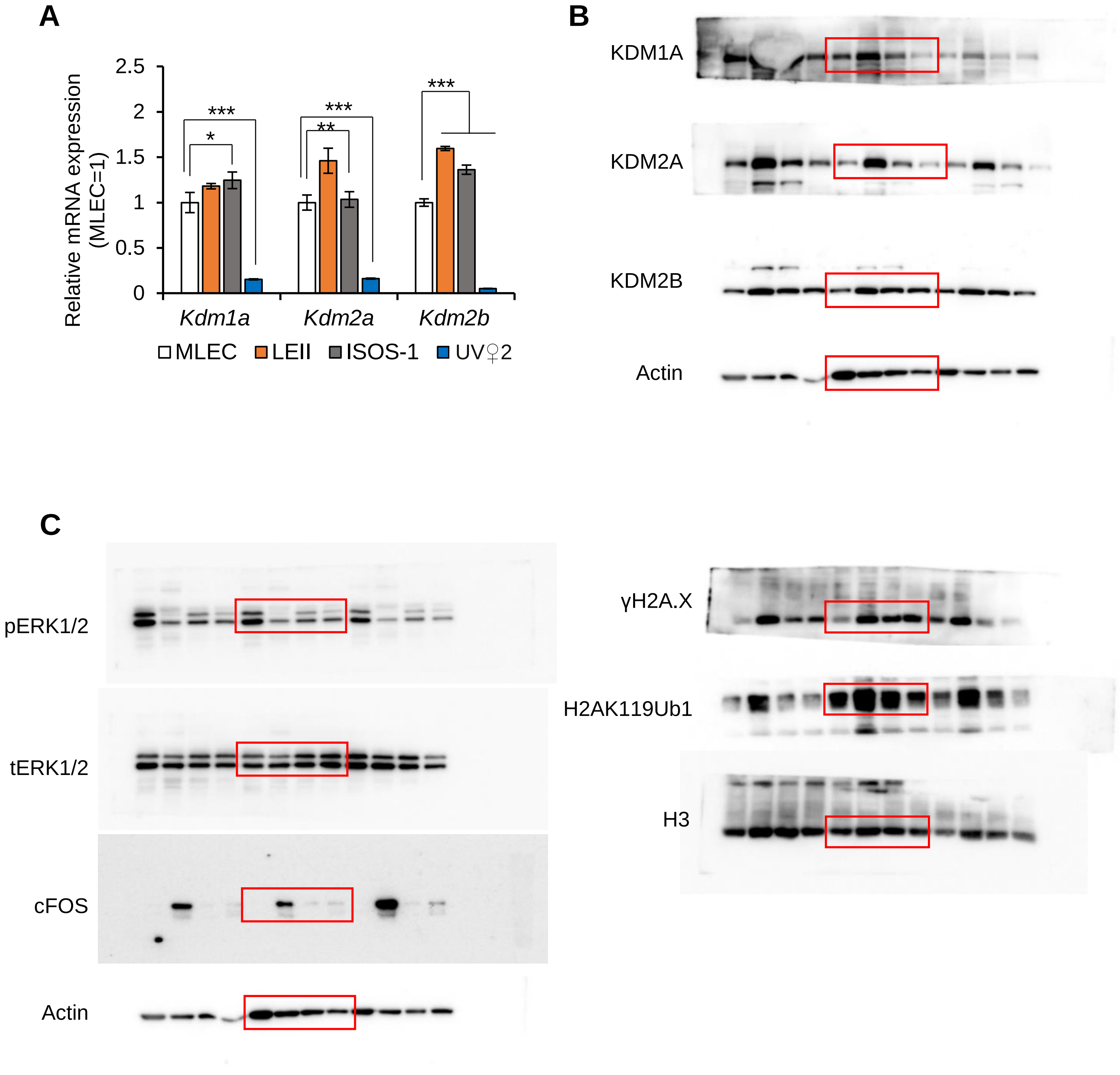

Supplementary figure 2. Molecular similarity of mouse HSA cell lines with canine HSA cell lines.
A, Relative gene expressions of *Kdm1a, Kdm2a,* and *Kdm2b* in mouse ISOS-1, LEII and UV♀2.. **B**, Uncropped images of Figure 1B. **C**. Uncropped images of Figure 1F.
Red boxes indicate the cropped area for Figure 1B and 1F. Data are presented as mean values ± s.d. ** *P*< 0.01*** *P*< 0.001, Tukey's test

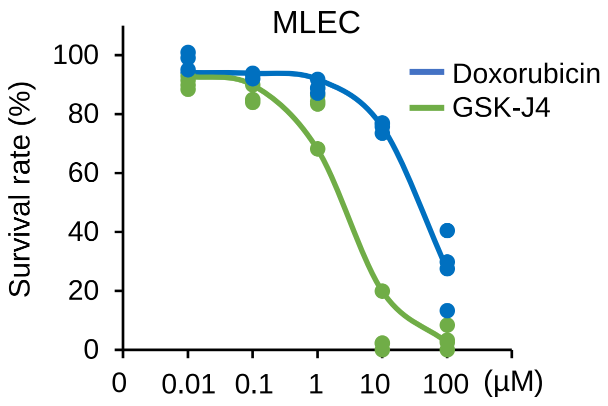

Drug	IC_{50}
Doxorubicin	77.39 μM
GSK-J4	5.06 μM

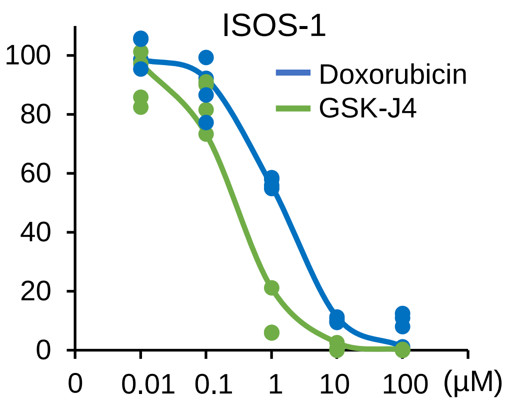

Drug3	IC_{50}
Doxorubicin	2.56 μM
GSK-J4	0.53 μM

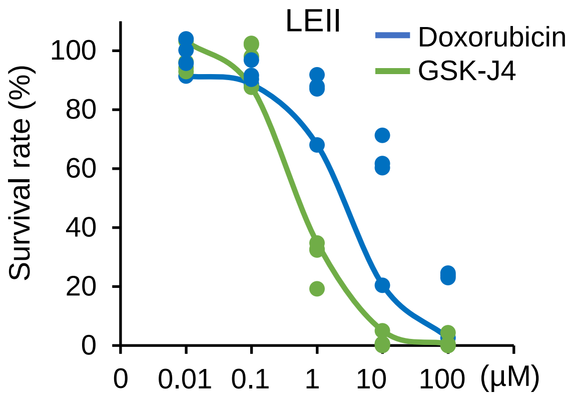

Drug	IC_{50}
Doxorubicin	5.25 μM
GSK-J4	1.04 μM

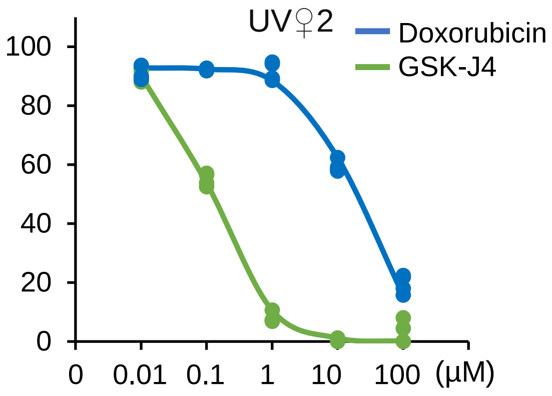

Drug	IC_{50}
Doxorubicin	38.05 μM
GSK-J4	0.24 μM

Supplementary figure 3. GSK-J4 efficacy on mouse HSA cell lines.
Survival rates and IC_{50} values of doxorubicin- or GSK-J4-treated MLEC, ISOS-1, LEII and UV♀2.

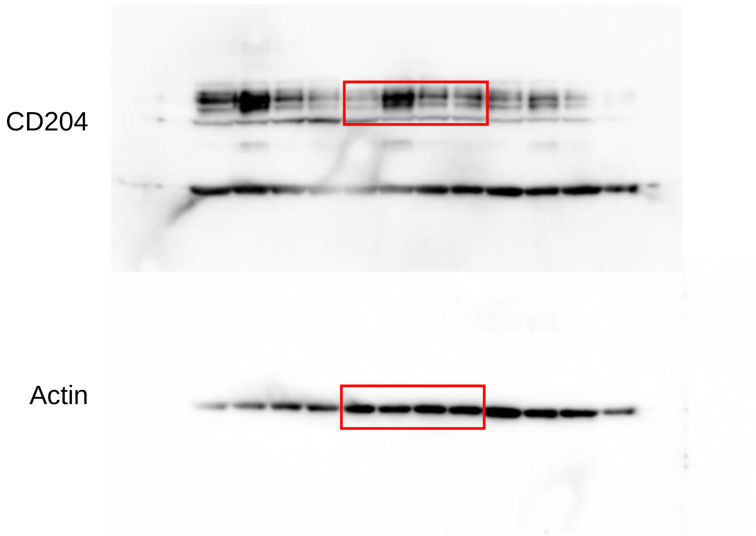

Supplementary figure 4. Uncropped images of Figure 2F. Red boxes indicate the cropped area for Figure 2F.

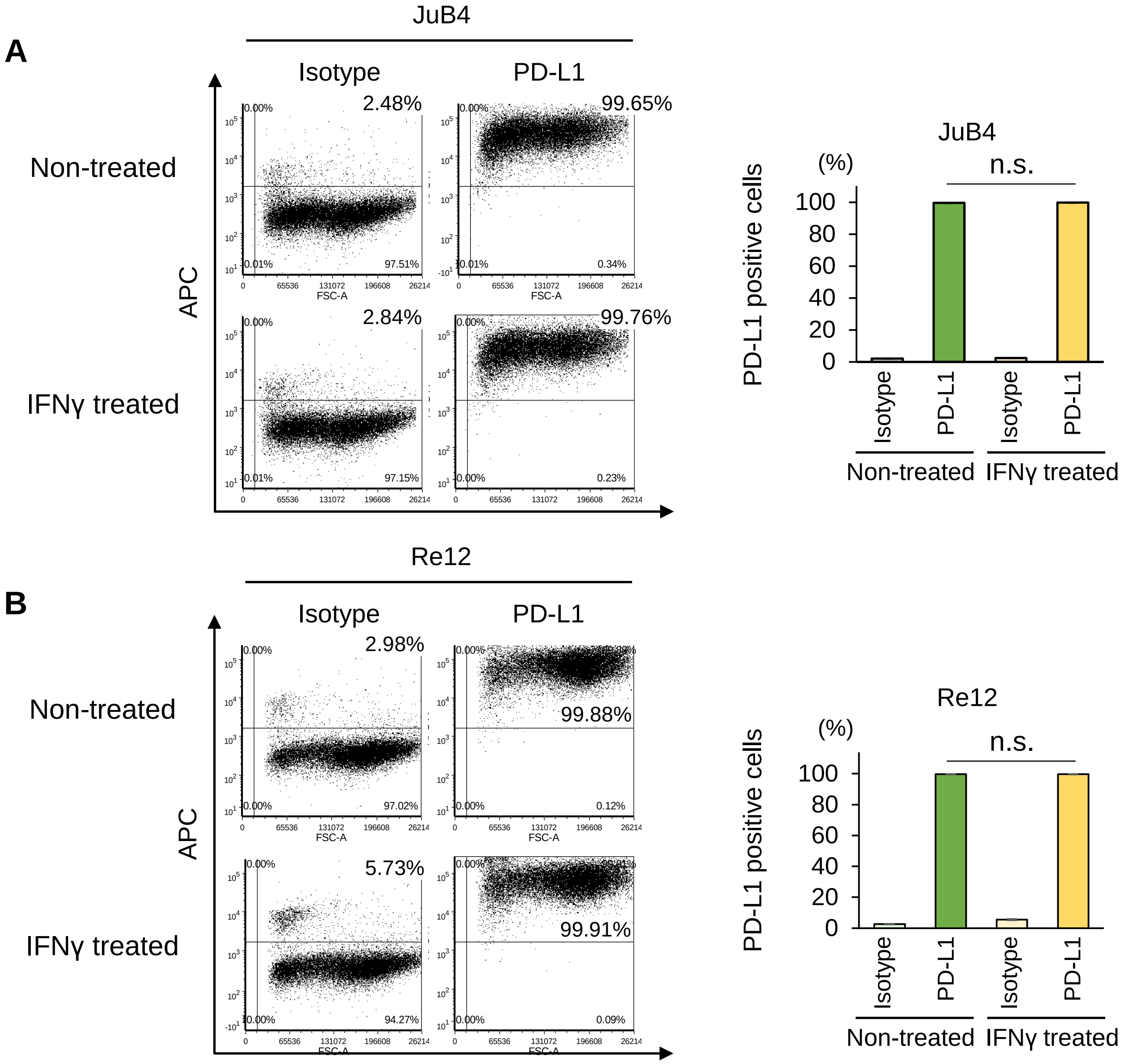

Supplementary figure 5. PD-L1 is constitutively expressed in canine HSA cell lines.
A and B, (Left) Representative images of flow cytometry for PD-L1 in JuB4 (A) and Re12 (B) cell lines with/without IFNγ. (Right) Quantitative analysis of the flow cytometry data. Data are presented as mean values ± s.d.

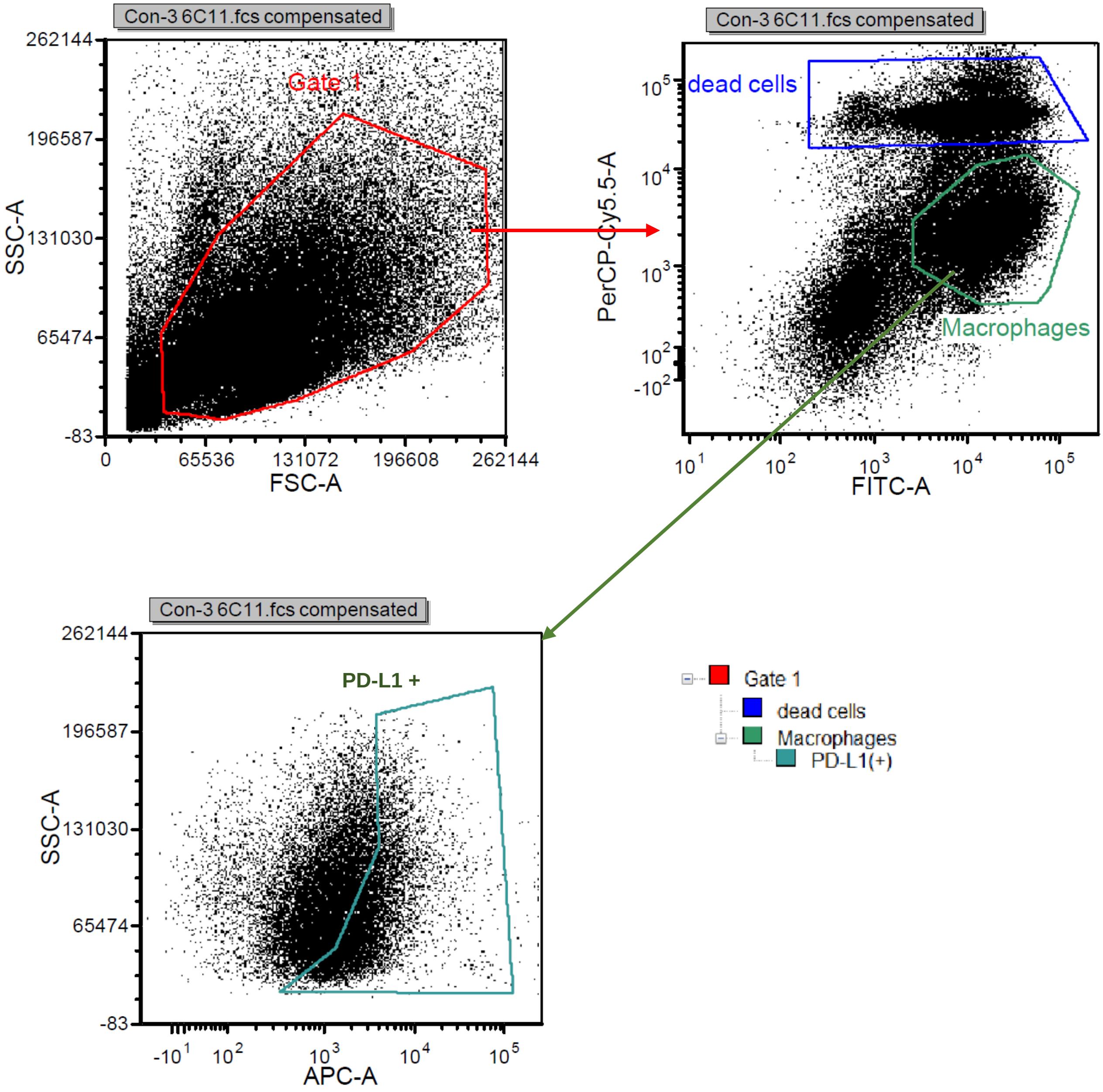

Supplementary figure 6. The gating method for PD-L1 analysis in peritoneal macrophages. Samples were stained with 7-AAD (PerCP-Cy5.5-A), F4/80 (FITC) and PD-L1 (APC). 7-AAD^{-}/F4/80^{+} population was selected as the live macrophage population.

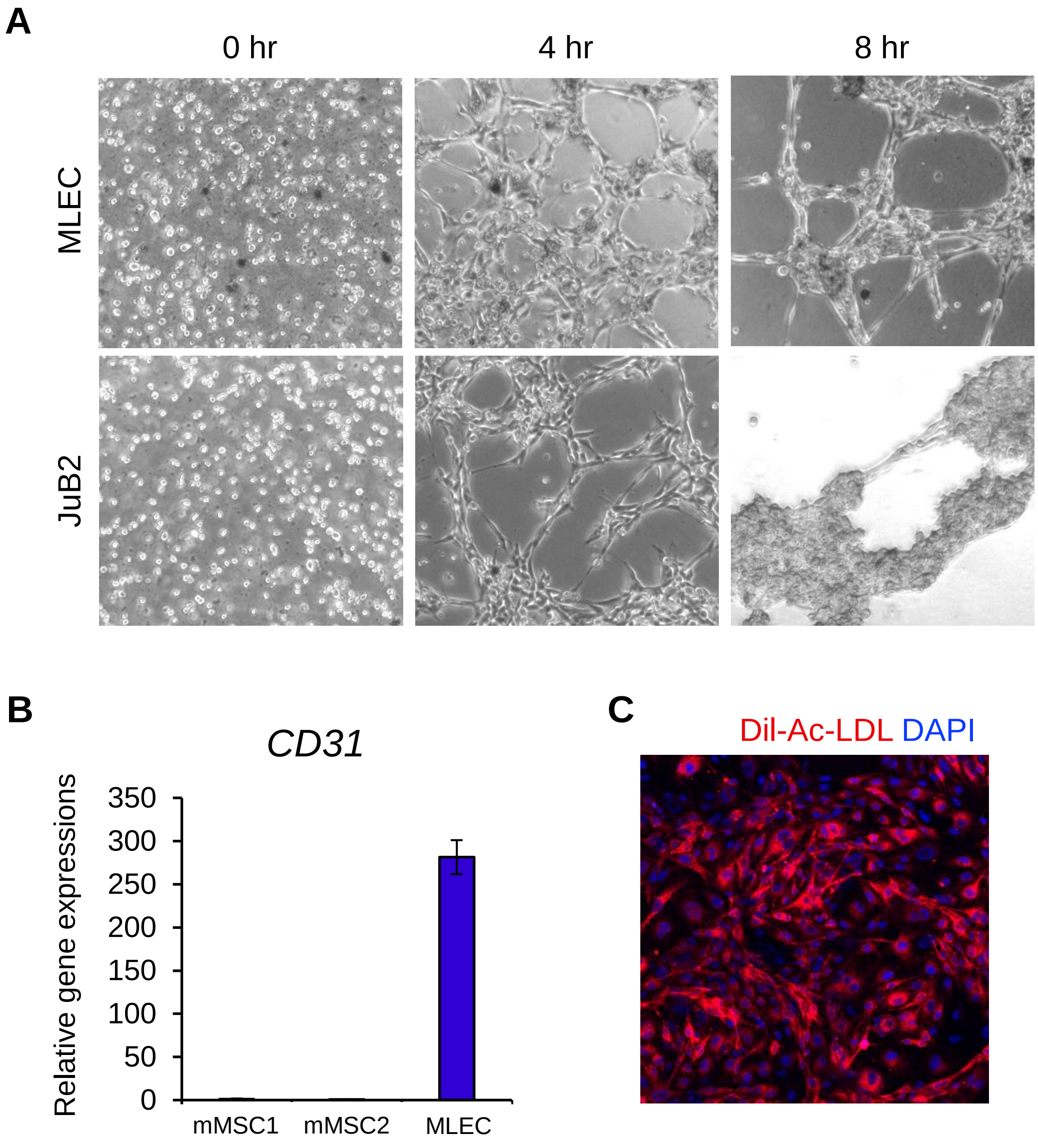

Supplementary figure 7. Verification and characterization of isolated MLEC.
A, Phase-contrast images of tube formation assay for MLEC and JuB2. **B,** Relative gene expression levels of *CD31* in mouse mesenchymal stem cells (mMSC) and MLEC. **C,** Dil-Ac-LDL uptake assay in isolated MLEC. Data are presented as mean values ± s.d.

Supplementary table. Primers used in this study.

Species	Target	Sequence
Mouse	Kdm1a (F)	TTCCCAGACATCATCAGTGG
	Kdm1a (R)	CCAGCCATAACTGCAATGTG
	Kdm2a (F)	ACTGCATAACCAACCGATCC
	Kdm2a (R)	CCTTCCTCATCACCATTTCC
	Kdm2b (F)	TCCTGCATAGCTTCAACGTG
	Kdm2b (R)	TAACGGAACTTGGGCTGAAC
	Tbp (F)	AACAGCCTTCCACCTTATGC
	Tbp (R)	AAGATGGGAATTCCAGGAGTC
	CD31 (F)	CCAAAGCCAGTAGCATCATGGTC
	CD31 (R)	GGATGGTGAAGTTGGCTACAGG
	IL-6 (F)	TACCACTTCACAAGTCGGAGGC
	IL-6 (R)	CTGCAAGTGCATCATCGTTGTTC
	Nos2 (F)	GAGACAGGGAAGTCTGAAGCAC
	Nos2 (R)	CCAGCAGTAGTTGCTCCTCTTC
	Tnfα (F)	GGTGCCTATGTCTCAGCCTCTT
	Tnfα (R)	GCCATAGAACTGATGAGAGGGAG
	Havcr2 (F)	ACAGACACTGGTGACCCTCCAT
	Havcr2 (R)	CAGCAGAGACTCCCACTCCAAT
	CD163 (F)	GGCTAGACGAAGTCATCTGCAC
	CD163 (R)	CTTCGTTGGTCAGCCTCAGAGA
	PD-L1 (F)	TGCGGACTACAAGCGAATCACG
	PD-L1 (R)	CTCAGCTTCTGGATAACCCTCG
